# Possible vascular ablation effect in the MR-guided HIFU treatment of uterine fibroids: description of an unexpected or desired effect?

**DOI:** 10.1186/2050-5736-3-S1-P90

**Published:** 2015-06-30

**Authors:** Felipe Shoiti Urakawa, Marcos Roberto de Menezes, Mauricio Moura, Stephanie Castro

**Affiliations:** 1 Institute of Cancer of São Paulo, São Paulo, Brazil

## Background/introduction

Ablation by MR guided high intensity focused ultrasound has become one of the main non-invasive methods for the treatment of symptomatic uterine fibroids, since 2004, when it was approved by the FDA. Much of this growing preference, due to its effectiveness in relieving symptoms, low complication rates, lack of ionizing radiation and prompt recovery after the procedure. Despite all these advantages, the procedure time has become a major constraint in greater diffusion of this technique, especially in the treatment of large fibroids. Another major challenge coming up in the treatment of lesional margins, more susceptible to risks of injuries to surrounding structures, increasing the chances of under-treatment. Given the direct relationship between the non-perfused volume of post-therapy target lesion - which corresponds to the area not contrasted on MRI T1-weighted - and the relief of symptoms, the search for higher ablation zone as possible should always be the ultimate goal. At this time of search for solutions through multicentric experience, a particular vascular effect has been described in the treatment of fibroids for MR-guided HIFU. There has been a reported case of shrinking fibroid treated with MR-guided HIFU estimated at 98% in which only the periphery of the lesion had been treated.[1] In this case, the reduced perfusion in the all fibroid was attributed to the tissue ischemia and necrosis caused by the ablation of major peripheral vessels. In 2011, a new method of treatment of uterine fibroids based on vascular mapping by MR angiography and arterial ablation spot was proposed, getting good results in the two patients described.[2] Given this phenomenon observed in sporadic cases in the literature, our proposal is to describe findings that corroborate the vascular ablation effect in a retrospective observational study of 6 cases of treatment of uterine fibroids with MR-guided HIFU.

## Methods

Between February 2011 and March 2014, 36 patients in reproductive age with symptomatic uterine fibroids were subjected to treatment with MRI-HIFU. Six of these patients had in common, besides excellent results in improving symptoms, a peculiar behavior in contimages post-treatment, denoting a particular vascular ablation effect. This effect is characterized by greater non-perfused volume (NPV) than the area effectively treated fibroid, whose regular and well-defined margins coincide with the boundaries of the target lesion. Adds to this concept the simultaneous achievement of adjacent satellites not perfused fibroids, non-target lesions, however indirectly affected. This group was gathered through retrospective observation of this peculiar behavior of therapeutic response, identifying all patients underwent coincidentally the same planning sonication. This pattern consisted of transverse division of the fibroid into two equal parts, starting the sonications for the lateral aspects of the periphery of the caudal half, focusing consecutive sessions in the same region. Thus concentrated energy has already been deposited in the region to start the next sonication, avoiding random sonications. Immediately after treatment, post-contrast T1-weighted sequences for evaluation of treatment outcome were performed. Such images were used to calculate the volume of fibroid and not perfused volume (NPV). The volumes of fibroids and NPVs were calculated by a method sectional sum. Regions of interest were outlined in each sectional image through a MR workstation and the volume of each segment was calculated and summed. The NPV is also calculated as a percentage of the volume of the fibroid to indicate the percentage ablated. Patients were followed 6-12 months after treatment with a questionnaire to assess symptoms and quality of life (Uterine Fibroid Symptom and Quality of Life) and magnetic resonance imaging to quantify shrinkage of fibroid volumes.

## Results and conclusions

In 6 patients, 9 fibroids were treated with the same pattern of sonication, focusing initially on the periphery of the inferior aspect of the fibroid. MR- HIFU. The treatment resulted in almost total fibroids desvascularization with nonperfused volume greater than 90%. In all cases was observed a peculiar behavior in control images post-treatment, characterized by greater non-perfused volume (NPV) than the area effectively treated fibroid, whose regular and well-defined margins coincide with the boundaries of the target lesion, and simultaneous adjacent satellites not perfused fibroids (non-target lesions).

These findings contained in the largest series of cases already described in the literature, reinforce a pattern of therapeutic response, pointing to a possible vascular ablation effect, idea raised sporadically in the literature. Obtaining this pattern of response, with adequate pre-treatment planning, has shown excellent results in control images post-ablation and major impact on improving the quality of life of patients.
